# The Thermococcales as a model system: historical perspectives and emerging tools

**DOI:** 10.1128/jb.00409-25

**Published:** 2025-11-05

**Authors:** Alina M. Galyon, Haruyuki Atomi, Thomas J. Santangelo

**Affiliations:** 1 Department of Biochemistry and Molecular Biology, Colorado State University 548293 , Fort Collins, Colorado, USA; 2 Department of Synthetic Chemistry and Biological Chemistry, Graduate School of Engineering, Kyoto, Japan; 3 Integrated Research Center for Carbon Negative Science, Institute of Advanced Energy, Kyoto University 34807, Uji, Japan; Geisel School of Medicine at Dartmouth, Hanover, New Hampshire, USA

**Keywords:** Archaea, *Thermococcus*, *Pyrococcus*, *Palaeococcus*, thermophile, hyperthermophile

## Abstract

Thermococcales are among the most widely studied hyperthermophilic Archaea and have become key models for understanding life at extreme temperatures. Early work in the 1980s culminated in the isolation of novel Thermococcales species from hydrothermal vents that grew rapidly, tolerated extreme heat, and metabolized diverse substrates, making them uniquely amenable for laboratory studies. Their thermostable enzymes and emerging genetic tools facilitated detailed investigations of core processes such as DNA replication, repair, and transcription under conditions that challenge most life forms. These practical advantages, together with the accumulation of tools and protocols, cemented the role of Thermococcales as a model system. Here, we recount how chance discoveries, environmental adaptations, and experimental practicality intersected to establish Thermococcales as a central model for studying archaeal biology and extremophile physiology.

## HISTORY AND DISCOVERY

The exploration of life in extreme environments gained prominence with Thomas Brock’s “Life at High Temperatures” (1), and his subsequent isolation of *Thermus aquaticus* with Hudson Freeze in 1969 (2) is often credited with introducing thermophilic (heat-loving) microbes to a broad audience. While Brock focused on hot-spring-tolerant thermophilic bacteria, many descriptions of extremophilic life predate his work (3, 4), laying a foundation for today’s expansive research on organisms thriving under conditions of extreme pressure, pH, salinity, and temperature. An ever-growing body of literature continues to describe and classify life into categories such as piezophiles/barophiles (pressure-loving), acidophiles/alkaliphiles (pH extremes), halophiles (salinity), thermophiles (up to ~80°C), and hyperthermophiles (>80°C). Polyextremophiles, such as piezophilic hyperthermophiles, have also been characterized with increasing frequency (5–9). Alongside the pioneering descriptions and isolations of extremophiles, the 1960s saw microbiology reshaped by seminal manuscripts that defined not only eukaryotes and prokaryotes (10) but that laid the foundation—first via substantial differences in physiology and morphology, and later via phylogeny—to warrant reclassification of known life into three domains. This latter concept was famously refined by Carl Woese and George Fox to define the Archaea in 1977, emerging from the phylogenetic analyses of 16S rRNA (11).

In the late 1970s and early 1980s, additional characteristic features were actively sought to support the revolutionary concept of a distinct domain. Some of the first systematic studies of archaeal biology were primarily driven by phylogenetic queries rather than on the basis of thermophily. Among these early investigations, Wolfram Zillig and Karl Stetter initially sought to discover novel Archaea and learn more about DNA-dependent RNA polymerases in the few archaeal species identified at the time, to better understand archaeal phylogeny. Assisted by Peter Palm and Sigfried Wunderl, their early sampling expeditions in 1980 to Iceland yielded novel hyperthermophilic Archaea, including the Thermoproteales, which Stetter and colleagues described in 1981 (12).

Among the earliest and still most widely studied archaeal hyperthermophiles is the order Thermococcales, which currently includes three genera: *Pyrococcus*, *Thermococcus*, and *Palaeococcus*. The founding member of the Thermococcales (*Thermococcus celer*) was discovered in 1982 by Wolfram Zillig from samples taken by Karl Stetter during a trip to Vulcano, Italy, marking the beginning of a novel field of archaeal research (13). Shortly thereafter, Gerhard Fiala, then a PhD student of Karl Stetter, isolated *Pyrococcus furiosus* (“raging fireball”), the first member of the Thermococcales with an optimal growth temperature of 100°C and a maximum of 104°C (14). The rapid growth (37-minute doubling time) to high cell densities at an optimal temperature of 100°C and its strong motility in defined media provided the moniker for “furiosus,” meaning raging.

Thermococcales quickly gained prominence due to their biotechnological potential, exemplified by the isolation and commercialization of thermostable high-fidelity DNA polymerases, such as “Vent DNA polymerase” from *Thermococcus litoralis* in 1990 (15), and the debut of *Pfu* DNA polymerase from *Pyrococcus furiosus* in 1991 (16). Considering the potential for many additional and valuable thermostable enzymes, the continued use of thermostable homologs to facilitate crystallographic structural determinations, and the ability to consume a wide range of substrates (17–20), including α-linked carbohydrates (21), *P. furiosus* and the Thermococcales quickly captured the attention of the scientific community. The discovery of its ability to grow on maltose stemmed from a Stetter lab party in a Bavarian beer garden, where Gerhard Fiala was teased that his novel “multi-substrate” strain (*P. furiosus*) would never grow on beer. Intrigued, he returned to the lab that same evening with a bottle in hand, and by the next morning, he discovered robust growth. The secret, later revealed, was maltose—a major component of beer and a preferred energy source for *P. furiosus*—in what was surely an amusing delight to the Germans, who likely raised a celebratory “Prost!” at the discovery.

While many Archaea occupy rather conventional niches in modest environments (a fact only recognized much later after their initial discovery), the conservation of typical eukaryotic-like information processing machinery in all Archaea coupled with many foundational biological queries of life at high temperatures fostered rapid investigations of hyperthermophiles. One major theme of research was driven by questions about how these temperature-resilient microbes maintain the stability of biomolecules in conditions previously thought to be detrimental to macromolecular integrity (22–34). The biotechnological advantages of enzymes derived from extremophiles were a second pillar of intrigue (35), and thermostable enzymes were undoubtedly a driving force for emergence of PCR in the mid-1980s (36). While thermostable DNA polymerases perhaps dominate the utility of hyperthermophilic enzymology in the laboratory, the utility of thermostable enzymes, in bulk, for paper and textile processing, biomass deconstruction, and greener alternatives to chemical conversions feed continued investigations of thermotolerance and hyperthermophilic biology (37–40). The upper-temperature limit of life has been pushed above 100°C, but the most widely studied (hyper)thermophiles more regularly thrive in the range of ~70°C–98°C (at one atmosphere) (41–43).

Early research on *P. furiosus* (and other members of the newly defined Thermococcales) was not without its challenges. Most existing laboratory equipment was not amenable to sustained use at extreme temperatures, and therefore, new technologies had to be developed to maintain anaerobic hyperthermophilic environments. The preferential use of elemental sulfur (S˚) for optimal growth and the subsequent production of massive quantities of hydrogen sulfide (H_2_S) provided real challenges for health (H_2_S is highly toxic), equipment maintenance (H_2_S is highly corrosive), and simple practical concerns (i.e., being a good neighbor and not flooding the building with noxious smelling gases). At such extreme temperatures, most conventional antimicrobials and antibiotics are unstable, raising concerns about cross-contamination between cultures (note that casual contaminations remain of low concern given the high-temperature incubations, but mixtures of hyperthermophiles remain a significant concern). Considerable innovations to permit growth on solid media, and genetic manipulations/ targeted gene disruption systems took another decade to develop. Curiously, although CRISPR systems are widely encoded within the Thermococcales (44, 45), and Archaea in general (46), and are well characterized and utilized for genetic manipulations (47), there appears to be an absence of Class II (single-subunit CRISPR-Cas systems including Types II (Cas9), V (Cas12), and VI (Cas13)) CRISPR systems in the hyperthermophilic Archaea (48)—a gap that may explain in part why these more user-friendly genome editing tools have not been directly adapted for genetic manipulation in Thermococcales.

Following the isolation of *P. furiosus*, the family Thermococcales has proven its utility with both biotechnological and foundational breakthroughs, revealed through characterization of a fascinating array of key species now divided into three distinct genera: *Palaeococcus*, *Pyrococcus*, and *Thermococcus*. Notably, the criteria used to classify many Thermococcales species into genera have been updated over time, resulting in changes to the genus of several species, e.g., *Pyrococcus kodakaraensis* was reclassified as *Thermococcus kodakaraensis* before an additional change to the spelling of the species to *kodakarensis* (49). The current members of the genus *Pyrococcus* are known exclusively from marine isolates, often thriving at great depths, and all with optimal growth temperatures approaching ~100°C. Key species within this group, such as *P. furiosus*, *P. abyssi*, and *P. horikoshii*, have emerged as crucial model organisms for investigating life in extreme conditions, offering invaluable insights into the resilience and adaptability of life on Earth, and sparking interest in the intriguing possibility that extremophilic environments on other planets and moons may harbor life. The genus *Palaeococcus*, typically thriving at a wider but lower temperature range (~60 and 88°C) than *Pyrococcus,* was first described in 2000 with the discovery of *Palaeococcus ferrophilus*, isolated from a black smoker chimney in the Ogasawara-Bonin Arc, Japan (50). Named for its ancient roots and utilization of iron, 16S rRNA sequencing showed that the *Palaeococcus* represent a lineage that likely diverged early in the evolution of the Thermococcales, perhaps preceding both *Thermococcus* and *Pyrococcus*. Most species described thus far within the order Thermococcales belong to the genus *Thermococcus*, boasting a rich diversity of freshwater and marine isolates that have been extensively characterized. Since the discovery of the first *Thermococcus—Thermococcus celer* (13)—many additional members of this genus have been identified, including *T. gammatolerans* (51)*, T. barophilus* (52), *T. onnurineus* (53), *T. nautilus* (54), *T. sibiricus* (55), *T. litoralis* (56), *T. chitonophagus* (57)*, T. alcaliphilus* (58), *T. acidaminovorans* (59), and *T. kodakarensis* (60), each offering unique insights into the adaptability and resilience of life at the limits of temperature. Fast-growing, easily isolated, high-titer, genetically accessible microbes encoding high-value commercial enzymology became a priority for the field, and the Archaea provided a new frontier for both microbiology and biotechnology.

## UNIQUE FEATURES, NOTABLE DISCOVERIES, AND FUTURE PERSPECTIVES OF THE THERMOCOCCALES

In 1998, *P. horikoshii* became the first member of the Thermococcales to have its full genome sequenced (61). This milestone paradoxically provided both key initial insights into how the Thermococcales likely adapt to extreme heat (and pressure), while also revealing an intriguing mystery: that nearly half of the genome (~1,000 genes) contain no obvious homologs outside of the Thermococcales or defined activities. Despite the availability of hundreds of complete Thermococcales genomes (9, 62–65), each ranging from ~1.5 to 3 Mbp in size, the function of more than ~30% of the predicted proteome is still unknown (66). Collective efforts to assign functions to these genes are progressing through the combined use of tools like AlphaFold and Dali-server for structural comparisons, coupled with experimental approaches such as the cloning, recombinant expression, and characterization of many proteins conserved within the Thermococcales. Moreover, the genetic definition of biosynthetic and catabolic pathways *in vivo* in the Thermococcales is slowly helping assign functions to genes of unknown function.

The basis for the distinct growth temperatures of *Palaeococcus* (~60°C), *Thermococcus* (~80°C), and *Pyrococcus* (~95°C) remains unclear, as does the absence of *Pyrococcus* species with intermediate optima (~80°C). Whether in-depth genome comparisons can provide insights into these differences remains an open question.

### Genetic systems

Within just five years of sequencing the first complete Thermococcales genome, and arguably the most advantageous overall milestone supporting the general prowess of the Thermococcales, a targeted gene disruption system was developed for *Thermococcus kodakaraensis* (*Tko*) (67). Derivations and refinements of this first system now permit precision modification of the genomes (including extrachromosomal elements) of many Thermococcales (68–73).

As genetic systems within the Thermococcales continue to evolve, scientists have leveraged these tools to define essential genes and gene function through targeted gene deletion and modifications. It is perhaps surprising to even mention temperature-sensitive phenotypes for the Thermococcales, but it is now clear that a number of individual mutations and deletions—encompassing distinct physiological traits from RNA modifications and stability (23, 25, 74), to lipid maturation and metabolism (75–77), to S-layer synthesis, DNA-dependent processes (78–85), and proper protein folding (86, 87)—are non-essential to model members of the Thermococcales but do confer the ability for many species to grow at the limits of their temperature range. The capacity to introduce sequences encoding affinity tags now allows for single-step purifications of stable, *in vivo*-associated protein complexes directly from host cells (88).

Current efforts aim to improve upon these genetic systems, focusing on increasing competency and overall transformation efficiency (89, 90). Such advancements will drive further exploration into Thermococcales biology, offering valuable insights into the unique adaptations that enable survival in extreme environments.

### Unique metabolic traits and mechanisms of energy conservation

Thermococcales utilize peptides and amino acids for catabolism, relying on 2-oxoacid: ferredoxin oxidoreductases (91–93) and NDP-forming acyl-CoA synthetases (94–96) to generate reduced ferredoxin and carry out substrate-level phosphorylation, respectively. Most Thermococcales also harbor a modified Embden-Meyerhof-Parnas (EMP) pathway (97–101) for glycolysis, highlighted by ADP- rather than ATP-dependent sugar kinases and glyceraldehyde-3-phosphate:ferredoxin oxidoreductase rather than an NAD-dependent dehydrogenase. A unique hallmark of Thermococcales metabolism is the preferential reduction of S˚ as the terminal electron acceptor by a membrane-bound sulfane reductase (MBS) (102). These organisms can alternatively reduce protons in the absence of S˚ by a 14-subunit membrane-bound hydrogenase (MBH) to generate hydrogen gas (H_2_), a process that couples hydrogen production to proton gradient generation and subsequently to a sodium gradient via an Mrp-type Na^+^/H^+^ antiporter within the complex. After more than 35 years of attempts using X-ray crystallography, the first structure of the Pfu hydrogenase has now been resolved by cryoEM, providing long-awaited insights into the architecture and mechanism of this central respiratory complex (103). Based on the homology to MBH, MBS is also assumed to couple S˚ reduction to sodium gradient generation for ATP synthesis by a sodium-dependent A_1_A_0_ ATP synthase. Both membrane-bound respiratory complexes rely on three reduced iron-sulfur ferredoxin proteins—each with distinct roles in the cell—as the primary electron carriers, effectively connecting carbon-based metabolic processes with respiration (104). In minus sulfur conditions, these organisms can generate hydrogen gas (H_2_) at extraordinary rates, the discovery of which has sparked significant interest in biofuel production studies within the Thermococcales, typically through dark fermentation strategies (105, 106).

*Thermococcus onnurineus*, with its unusual capacity for lithotrophic growth on carbon monoxide (CO), using CO as an electron donor and protons (H^+^) as the terminal electron acceptor, has emerged as a promising candidate for renewable energy research, specifically for its application in bioreactors designed to convert industrial CO emissions—typically from steel manufacturing—into clean-burning H_2_ fuel (107–109).

Studies on the metabolism in Thermococcales have led to discoveries of previously unrecognized enzyme reactions and metabolic pathways. While ribulose-1,5-bisphosphate carboxylase-oxygenase (RuBisCO) is conventionally known to participate in the Calvin-Benson-Bassham (CBB) cycle in photosynthetic organisms, Thermococcales encode a unique archaeal form III RuBisCO that functions in the pentose bisphosphate pathway, enabling the cells to salvage nucleosides to nucleotides or direct their ribose moieties to central carbon metabolism (110–112). The ability of all members of the Thermococcales to catabolize peptides and amino acids may reflect the fact that amino acids display higher stability at high temperatures compared to sugars, particularly aldoses. The use of sodium gradients may reflect the fact that they are less leaky across the membrane compared to proton gradients at high temperature. Many marine species within this order also typically encode a wealth of chitinases, suggesting the use of chitin as a primary substrate in native environments (20, 113).

### Enzymology/structural discoveries

Extensive research into the catalytic versatility and robustness of Thermococcales enzymes under extreme conditions (extremozymes) has demonstrated their remarkable ability to function in environments characterized by extreme temperatures, salinity, and pH. Some Thermococcales enzymes even exhibit high activity in the face of temperature and chemical denaturants including 8 M urea (114). Thermococcales enzymes have thus become valuable models for engineering conventional enzymes to expand their biocatalytic limits and make them better suited for various industrial applications. The high-temperature environments in which many Thermococcales thrive are often characterized by high metal stoichiometry, with elevated concentrations of metals not typically found in other ecosystems (some *Pyrococcus* cells have even been found to contain trace quantities of lead and uranium). Therefore, it is not surprising that these organisms have evolved a unique metal biosignature in which their metalloproteins harness perhaps unexpected metal cofactors such as tungsten (115–117), cobalt (118), and molybdenum (119). The development of novel strategies and further research into the Thermococcales metalloproteome continues to reveal novel insights into the unusual ability of these organisms to mobilize metal ions from their environments (120).

The late 1990s and early 2000s marked a golden era of structural biology, presenting a unique opportunity for researchers to investigate how hyperthermophiles maintain protein stability at temperatures nearing or exceeding the boiling point of water. Advancements in tools such as X-ray crystallography and electron microscopy allowed for detailed structural analyses of Thermococcales proteins, generating visual insights into different cellular processes ranging from metabolism to DNA replication and transcription (117, 121–125). Interestingly, structural determination of Thermococcales proteins did not immediately reveal any singular, striking feature to explain thermostability; this challenged researchers to think beyond simplistic explanations and instead consider the complex interplay of structural and thermodynamic factors that enable proteins to function in extreme conditions (126, 127). Later advancements in cryo-EM technology further expanded our understanding of Thermococcales systems biology by revealing structural details at unprecedented resolution (128).

While the genomes of many Thermococcales have now been sequenced, analyses have revealed no significant changes in amino acid composition that directly account for protein thermostability—a stark contrast to key adaptations noted in protein content in halophilic species (22, 129). Although structural comparisons of homologous proteins deriving from mesophiles and (hyper)thermophiles have led to proposals that increased hydrophobicity in the protein core, higher numbers of salt bridges, and reduction of loop sizes result in higher thermostability, further determinants remain largely enigmatic. Moreover, the modest ~40%–50% GC content of most Thermococcales genomes underscores that genomic GC composition is not correlated with optimal growth temperature (62). Instead, the retention and abundance of many nucleoid-associated proteins, including bona fide histones (78, 130), together with the stability of topologically constrained DNA at temperatures up to 107°C (31), likely play critical roles in maintaining genomic and extrachromosomal DNA integrity under extreme conditions.

### Replication, recombination, and repair

Beyond their unique metabolic traits, many Thermococcales exhibit exceptional radiation tolerance. Notably, *Thermococcus gammatolerans*, isolated from hydrothermal chimneys rich in heavy metals, demonstrates an extraordinary ability to survive radiation doses hundreds of times higher than those typically encountered on Earth (131). Many Thermococcales species can withstand exposure to >3 kGy of radiation, levels exceeding the tolerance of even *Deniococcus radiotolerans*, with only minimal reductions in viability (132, 133). These results were obtained in rich media, which may contribute radioprotective effects (e.g., antioxidants and other scavengers present in yeast extract or peptides), so the extreme tolerance reported should be interpreted in that experimental context. Nonetheless, such resilience presents an intriguing opportunity to study robust DNA repair mechanisms, particularly pathways adept at rapidly addressing double-strand breaks (DSBs). It is peculiar that despite thriving in environments predicted to destabilize DNA and likely dramatically increase rates of DNA damage, there is very little evidence of Thermococcales specific (or even archaeal-specific DNA repair pathways to combat the predicted insults to genomic DNA (134, 135). Across the Thermococcales, with the exception of mobile genetic elements, there are only modest mutation rates (136), suggesting that continued research on *T. gammatolerans* and other members of the Thermococcales holds promise for advancements in bioremediation of radiation-contaminated sites and potential medical applications where enhanced DNA repair mechanisms could be harnessed for therapeutic benefits.

Like nearly all hyperthermophiles, the Thermococcales nearly universally encode for reverse gyrase—a specialized topoisomerase that introduces positive supercoils into DNA (137, 138). Although often described as increasing the melting temperature of DNA, experimental evidence indicates that positive supercoiling does not stabilize topologically constrained DNA beyond that of negative supercoiling, with both behaving similarly up to 107°C (31). This observation may help explain why certain hyperthermophiles, such as *Archaeoglobus* and *Thermoplasma*, which encode both a conventional DNA gyrase and reverse gyrase, maintain negatively supercoiled genomes (139, 140). More recently, the introduction of a thermophilic DNA gyrase into *T. kodakarensis* showed that extensive negative supercoiling did not alter its optimal growth temperature or viability but did induce negative supercoiling of resident plasmids (141). Interestingly, while reverse gyrase is dispensable for hyperthermophilic growth in the model species *T. kodakarensis,* its deletion results in temperature-sensitive growth, lowering the growth limit from ~95°C to just ~90°C (142), hinting at its likely importance in sustaining growth in extreme thermophilic conditions.

A notable preponderance of inteins (intervening proteins) (143), frequently invading critical proteins involved in replication, recombination, and DNA repair (144), has also been identified in Thermococcales genomes. Inteins have driven considerable research interest due to their implications for commercial ventures (145). For instance, the recombinant expression strategies, and therefore the commercial value of thermostable DNA polymerases, have been complicated by the frequent invasion of their encoding loci by inteins, necessitating a deep understanding of the fundamental process of intein excision (146). Many of the now common intein-based self-cleaving fusion protein purification strategies are a direct byproduct of the need to produce high-fidelity Thermococcales-derived DNA polymerases in recombinant hosts (147). Beyond this, inteins have become versatile tools for protein labeling, biosensing, and the construction of artificial protein switches, expanding the versatility of genetic and biochemical assays (148, 149).

The discovery of DNA polymerase D (PolD) in *Pyrococcus* challenged prevailing models of archaeal replication by revealing a novel, archaeal-specific D-family polymerase (150). Unlike the well-characterized A, B, C, and Y families, PolD was found to be structurally and evolutionarily distinct, serving as the primary replicative polymerase in many Thermococcales—contrary to earlier assumptions that PolB, a eukaryotic homolog, played this role (151). Structural analyses further underscored PolD’s uniqueness, revealing a double-psi beta-barrel (DPBB) fold typically seen in multi-subunit RNA polymerases rather than DNA polymerases (152), suggesting a possible evolutionary link between replication and transcription and prompting new questions about the origins of DNA replication machinery. Other major findings include the Holliday junction resolvase (Hjc) from *P. furiosus*. Hjc, which cleaves the Holliday junctions that occur in genetic recombination and double-strand break repair processes, is structurally distinct from the enzymes of bacteria and eukaryotes and is widespread in archaea (153). Hef (helicase-associated endonuclease for fork-structured DNA) was discovered in *P. furiosus* as an enzyme that resolves stalled DNA forks during DNA replication (154, 155). Afterward, the human Hef homolog was identified as a component of the Fanconi anemia core complex, which contributed to a better understanding of its function (156, 157).

Genetic studies within the Thermococcales have redefined replisome composition and replicative mechanisms, with the heterodimeric, archaeal-specific DNA polymerase D shown to be primarily responsible for replication in many Thermococcales that do not initiate at a defined origin (83, 151, 158–161). Early experimental identification of the origin of replication and its initiator protein in the Thermococcales member *Pyrococcus abyssi* (162, 163) further underscored the group’s central role in shaping our understanding of archaeal DNA replication. The polyploid nature of Thermococcales provides a critical advantage in their natural environments by harboring an ample reservoir of replicative and repair templates for homology-directed repair and replication (164). This feature enables them to swiftly resolve DNA damage and rebuild their genomes under conditions of high mutation pressure or DNA instability.

### Transcription and translation

The complete transcription cycle and activities of many factors that regulate the activities of the multisubunit RNA polymerase have been defined (165, 166), elucidating the mechanisms of initiation (167–170), elongation (123, 171–173), termination (81, 174–177), and the interactions with transcriptional regulators that modulate RNA polymerase activity (178, 179). Furthermore, significant progress has been made in understanding translation-based regulation of gene expression, including the modulation of mRNA stability and accessibility (180). Together, these insights have revealed the intricate coordination between transcription and translation—two processes that are coupled with one another—highlighting how these organisms fine-tune gene expression (181).

### Mobile genetic elements

Examination of Thermococcales genomes has revealed a striking disparity in the distribution of mobile genetic elements, with some species harboring an abundance while others display a near-absence (182–184). Notably, viral-host interactions have left a significant genomic footprint and have resulted in stable extrachromosomal elements (i.e., plasmids), expansion of CRISPR arrays (45), and the retention of both incapacitated and active viral genomes in the host genome (183, 185, 186). Many members of the Thermococcales possess extrachromosomal genetic elements that likely function to increase adaptability and resilience in extreme environments (182). Plasmids can be exchanged between species, and for some model members of the Thermococcales, their natural plasmids have been repurposed into sophisticated expression platforms that can be used to add new characteristics to strains through ectopic protein expression (187–191). Viruses (PAV1 in *P. abyssi* and TPV1 in *T. prieurii*) (192, 193), proviruses (TKV1 to TKV4 in *T. kodakarensis* and TGV1/TGV2 in *T. gammatolerans*) (66, 79), and virus-like vesicles (184, 194) have likely influenced the microbial community dynamics in which Thermococcales inhabit by mediating horizontal gene transfer and driving host genome evolution (186). Moreover, the interaction between viruses and their Thermococcales hosts has notably led to the evolution of defense mechanisms such as a diverse set of CRISPR systems, serving as an adaptive immune response to viral infections (44, 45, 47, 195).

### Small RNA and protein-based regulation

Thermococcales genomes harbor large numbers of small noncoding RNAs (sRNAs). These sRNAs are highly abundant and often display distinct expression patterns under various growth and stress conditions, emphasizing their likely critical roles in regulating gene expression, stress responses, and adaptation to extreme environments (196–201). Additionally, the unknown actions of small uncharacterized proteins—often encoded by short open reading frames—represent an intriguing yet underexplored research frontier as they likely participate in diverse cellular functions, such as stabilizing macromolecular complexes, modulating enzymatic activities, or mediating interactions with nucleic acids. Their small size and evolutionary plasticity suggest they may be critical regulators of cellular processes, particularly under the challenging conditions that define the Thermococcales niche. Further exploration of the dark side of the Thermococcales genome offers exciting potential for biotechnological applications.

### Evolutionary perspectives

The Thermococcales (and most Archaea) are particularly intriguing due to their hybrid biochemical features. Many Thermococcales enzymes that direct central metabolism are either unique to Archaea or homologous to bacterial systems (66, 202), whereas the central information processing machinery (i.e., replication, recombination, repair, transcription, and portions of translation) exhibits remarkable similarities with their eukaryotic counterparts (134, 135, 203). The Thermococcales, and Archaea in general, also provide a unique opportunity to explore the “lipid divide”: the retention of ether-linked isoprenoid-derived lipids in their cellular membranes in contrast to the fatty-acid-derived, ester-linked lipids in membranes of Bacteria and Eukarya (204). Phylogenetic analyses consistently place the Thermococcales on some of the earliest and deepest branches of the tree of life (205, 206), suggesting shared traits with ancient organisms.

Earlier work proposed that the Last Universal Common Ancestor (LUCA) may have been a hyperthermophile, but more recent evidence suggests otherwise as LUCA likely lacked reverse gyrase—a gene conserved in all known organisms growing above ~80°C (207). In contrast, the Last Archaeal Common Ancestor (LACA) has long been thought to have been a hyperthermophile, with Thermococcales representing a lineage that maintained this lifestyle in a continuously hot environment. However, the discovery of mesophilic close relatives of the Thermococcales, including the Theionarchaea and Methanofastidiosa, which have been grouped with the Thermococci in the clade *Acherontia* (208), complicates this picture. These findings raise the possibility that Thermococcales may have evolved from a mesophilic last common *Acherontia* ancestor through adaptation to hyperthermophily. The opposite scenario—that Thermococcales retained hyperthermophily from a hot-adapted ancestor—remains equally plausible. This “50/50” uncertainty highlights an intriguing evolutionary story and underscores the importance of future studies aimed at disentangling whether hyperthermophily in Thermococcales represents an ancestral state or a derived adaptation.

## CONCLUSION

The Thermococcales have transitioned from initial curiosities of extreme environments to genetically accessible model organisms that continue to yield critical insights into archaeal biology, extremophile physiology, and the evolutionary history of core cellular processes. The robust enzymology, unusual metabolic traits, and hybrid features of the Thermococcales spanning all domains of life continue to inform foundational questions in molecular evolution, cellular resilience, and the origins of informational machinery. Ongoing efforts to assign functions to the many genes of unknown activity, improve genetic systems, and decode the roles of small RNAs, inteins, and viral elements will further elevate the utility of Thermococcales in both fundamental and applied science. With large portions of their genomes still uncharacterized and regulatory layers such as small RNAs and virus-host dynamics only beginning to be defined, continued work in this lineage is likely to expand both our fundamental understanding of archaeal cell biology and the toolkit for high-temperature biotechnology.

## References

[1] Brock TD. 1967. Life at high temperatures. Science 158:1012–1019. doi:10.1126/science.158.3804.1012 4861476

[2] Brock TD, Freeze H. 1969. Thermus aquaticus gen. n. and sp. n., a nonsporulating extreme thermophile. J Bacteriol 98:289–297. doi:10.1128/jb.98.1.289-297.1969 5781580 PMC249935

[3] Reiser R, Tasch P. 1960. Investigation of the viability of osmophile bacteria of great geological age. Trans Kans Acad Sci 63:31–34. doi:10.2307/3626919 13740659

[4] Dombrowski H. 1963. Bacteria from paleozoic salt deposits. Ann N Y Acad Sci 108:453–460. doi:10.1111/j.1749-6632.1963.tb13400.x 14028514

[5] Seckbach J, Oren A, Stan-Lotter H. 2013. Polyextremophiles: life under multiple forms of stress. Springer Science & Business Media.

[6] Dalmasso C, Oger P, Selva G, Courtine D, L’Haridon S, Garlaschelli A, Roussel E, Miyazaki J, Reveillaud J, Jebbar M, Takai K, Maignien L, Alain K. 2016. Thermococcus piezophilus sp. nov., a novel hyperthermophilic and piezophilic archaeon with a broad pressure range for growth, isolated from a deepest hydrothermal vent at the Mid-Cayman Rise. Syst Appl Microbiol 39:440–444. doi:10.1016/j.syapm.2016.08.003 27638197

[7] Zeng X, Birrien J-L, Fouquet Y, Cherkashov G, Jebbar M, Querellou J, Oger P, Cambon-Bonavita M-A, Xiao X, Prieur D. 2009. Pyrococcus CH1, an obligate piezophilic hyperthermophile: extending the upper pressure-temperature limits for life. ISME J 3:873–876. doi:10.1038/ismej.2009.21 19295639

[8] Birrien J-L, Zeng X, Jebbar M, Cambon-Bonavita M-A, Quérellou J, Oger P, Bienvenu N, Xiao X, Prieur D. 2011. Pyrococcus yayanosii sp. nov., an obligate piezophilic hyperthermophilic archaeon isolated from a deep-sea hydrothermal vent. Int J Syst Evol Microbiol 61:2827–2881. doi:10.1099/ijs.0.024653-0 21239564

[9] Vannier P, Marteinsson VT, Fridjonsson OH, Oger P, Jebbar M. 2011. Complete genome sequence of the hyperthermophilic, piezophilic, heterotrophic, and carboxydotrophic archaeon Thermococcus barophilus MP. J Bacteriol 193:1481–1482. doi:10.1128/JB.01490-10 21217005 PMC3067617

[10] Stanier RY, Niel CB. 1962. The concept of a bacterium. Archiv Mikrobiol 42:17–35. doi:10.1007/BF00425185 13916221

[11] Woese CR, Fox GE. 1977. Phylogenetic structure of the prokaryotic domain: the primary kingdoms. Proc Natl Acad Sci USA 74:5088–5090. doi:10.1073/pnas.74.11.5088 270744 PMC432104

[12] Zillig W, Stetter KO, Schäfer W, Janekovic D, Wunderl S, Holz I, Palm P. 1981. Thermoproteales: a novel type of extremely thermoacidophilic anaerobic archaebacteria isolated from Icelandic solfataras. Syst Appl Microbiol 2:205–227. doi:10.1016/S0721-9571(81)80001-4

[13] Zillig W, Holz I, Janekovic D, Schäfer W, Reiter WD. 1983. The archaebacterium Thermococcus celer represents, a novel genus within the thermophilic branch of the archaebacteria. Syst Appl Microbiol 4:88–94. doi:10.1016/S0723-2020(83)80036-8 23196302

[14] Fiala G, Stetter KO. 1986. Pyrococcus furiosus sp. nov. represents a novel genus of marine heterotrophic archaebacteria growing optimally at 100°C. Arch Microbiol 145:56–61. doi:10.1007/BF00413027

[15] Kong H, Kucera RB, Jack WE. 1993. Characterization of a DNA polymerase from the hyperthermophile archaea Thermococcus litoralis. Vent DNA polymerase, steady state kinetics, thermal stability, processivity, strand displacement, and exonuclease activities. J Biol Chem 268:1965–1975. doi:10.1016/S0021-9258(18)53949-1 8420970

[16] Lundberg KS, Shoemaker DD, Adams MWW, Short JM, Sorge JA, Mathur EJ. 1991. High-fidelity amplification using a thermostable DNA polymerase isolated from Pyrococcus furiosus. Gene 108:1–6. doi:10.1016/0378-1119(91)90480-y 1761218

[17] Jorgensen S, Vorgias CE, Antranikian G. 1997. Cloning, sequencing, characterization, and expression of an extracellular α-amylase from the hyperthermophilic archaeon Pyrococcus furiosus in Escherichia coli and Bacillus subtilis. J Biol Chem 272:16335–16342. doi:10.1074/jbc.272.26.16335 9195939

[18] Koning SM, Elferink MGL, Konings WN, Driessen AJM. 2001. Cellobiose uptake in the hyperthermophilic archaeon Pyrococcus furiosus is mediated by an inducible, high-affinity ABC transporter. J Bacteriol 183:4979–4984. doi:10.1128/JB.183.17.4979-4984.2001 11489849 PMC95372

[19] Lee H-S, Shockley KR, Schut GJ, Conners SB, Montero CI, Johnson MR, Chou C-J, Bridger SL, Wigner N, Brehm SD, Jenney FE, Comfort DA, Kelly RM, Adams MWW. 2006. Transcriptional and biochemical analysis of starch metabolism in the hyperthermophilic archaeon Pyrococcus furiosus. J Bacteriol 188:2115–2125. doi:10.1128/JB.188.6.2115-2125.2006 16513741 PMC1428126

[20] Tanaka T, Fujiwara S, Nishikori S, Fukui T, Takagi M, Imanaka T. 1999. A unique chitinase with dual active sites and triple substrate binding sites from the hyperthermophilic archaeon Pyrococcus kodakaraensis KOD1. Appl Environ Microbiol 65:5338–5344. doi:10.1128/AEM.65.12.5338-5344.1999 10583986 PMC91726

[21] Schäfer T, Schönheit P. 1992. Maltose fermentation to acetate, CO_2_ and H_2_ in the anaerobic hyperthermophilic archaeon Pyrococcus furiosus: evidence for the operation of a novel sugar fermentation pathway. Arch Microbiol 158:188–202. doi:10.1007/BF00290815

[22] Reed CJ, Lewis H, Trejo E, Winston V, Evilia C. 2013. Protein adaptations in archaeal extremophiles. Archaea 2013:373275. doi:10.1155/2013/373275 24151449 PMC3787623

[23] Fluke KA, Fuchs RT, Tsai Y-L, Talbott V, Elkins L, Febvre HP, Dai N, Wolf EJ, Burkhart BW, Schiltz J, Brett Robb G, Corrêa IR, Santangelo TJ. 2024. The extensive m^5^C epitranscriptome of Thermococcus kodakarensis is generated by a suite of RNA methyltransferases that support thermophily. Nat Commun 15:7272. doi:10.1038/s41467-024-51410-w 39179532 PMC11344067

[24] Gomes-Filho JV, Randau L. 2019. RNA stabilization in hyperthermophilic archaea. Ann N Y Acad Sci 1447:88–96. doi:10.1111/nyas.14060 30994930

[25] Orita I, Futatsuishi R, Adachi K, Ohira T, Kaneko A, Minowa K, Suzuki M, Tamura T, Nakamura S, Imanaka T, Suzuki T, Fukui T. 2019. Random mutagenesis of a hyperthermophilic archaeon identified tRNA modifications associated with cellular hyperthermotolerance. Nucleic Acids Res 47:1964–1976. doi:10.1093/nar/gky1313 30605516 PMC6393311

[26] Shigi N, Suzuki T, Terada T, Shirouzu M, Yokoyama S, Watanabe K. 2006. Temperature-dependent biosynthesis of 2-thioribothymidine of Thermus thermophilus tRNA. J Biol Chem 281:2104–2113. doi:10.1074/jbc.M510771200 16317006

[27] Panja AS, Maiti S, Bandyopadhyay B. 2020. Protein stability governed by its structural plasticity is inferred by physicochemical factors and salt bridges. Sci Rep 10:1822. doi:10.1038/s41598-020-58825-7 32020026 PMC7000726

[28] Tomikawa C, Yokogawa T, Kanai T, Hori H. 2010. N^7^-Methylguanine at position 46 (m^7^G46) in tRNA from Thermus thermophilus is required for cell viability at high temperatures through a tRNA modification network. Nucleic Acids Res 38:942–957. doi:10.1093/nar/gkp1059 19934251 PMC2817472

[29] Kowalak JA, Dalluge JJ, McCloskey JA, Stetter KO. 1994. The role of posttranscriptional modification in stabilization of transfer RNA from hyperthermophiles. Biochemistry 33:7869–7876. doi:10.1021/bi00191a014 7516708

[30] D’Amico S, Marx J-C, Gerday C, Feller G. 2003. Activity-stability relationships in extremophilic enzymes. J Biol Chem 278:7891–7896. doi:10.1074/jbc.M212508200 12511577

[31] Marguet E, Forterre P. 1994. DNA stability at temperatures typical for hyperthermophiles. Nucleic Acids Res 22:1681–1686. doi:10.1093/nar/22.9.1681 8202372 PMC308049

[32] Lurz R, Grote M, Dijk J, Reinhardt R, Dobrinski B. 1986. Electron microscopic study of DNA complexes with proteins from the archaebacterium Sulfolobus acidocaldarius. EMBO J 5:3715–3721. doi:10.1002/j.1460-2075.1986.tb04705.x 16453745 PMC1167416

[33] Sandman K, Krzycki JA, Dobrinski B, Lurz R, Reeve JN. 1990. HMf, a DNA-binding protein isolated from the hyperthermophilic archaeon Methanothermus fervidus, is most closely related to histones. Proc Natl Acad Sci USA 87:5788–5791. doi:10.1073/pnas.87.15.5788 2377617 PMC54413

[34] Bouthier de la Tour C, Portemer C, Huber R, Forterre P, Duguet M. 1991. Reverse gyrase in thermophilic eubacteria. J Bacteriol 173:3921–3923. doi:10.1128/jb.173.12.3921-3923.1991 1646792 PMC208029

[35] Adams MWW. 1993. Enzymes and proteins from organisms that grow near and above 100°C. Annu Rev Microbiol 47:627–658. doi:10.1146/annurev.mi.47.100193.003211 8257111

[36] Chien A, Edgar DB, Trela JM. 1976. Deoxyribonucleic acid polymerase from the extreme thermophile Thermus aquaticus. J Bacteriol 127:1550–1557. doi:10.1128/jb.127.3.1550-1557.1976 8432 PMC232952

[37] Kumar V, Marín-Navarro J, Shukla P. 2016. Thermostable microbial xylanases for pulp and paper industries: trends, applications and further perspectives. World J Microbiol Biotechnol 32:34. doi:10.1007/s11274-015-2005-0 26754672

[38] Turner P, Mamo G, Karlsson EN. 2007. Potential and utilization of thermophiles and thermostable enzymes in biorefining. Microb Cell Fact 6:9. doi:10.1186/1475-2859-6-9 17359551 PMC1851020

[39] Stamford TLM, Stamford NP, Coelho L, Araújo JM. 2001. Production and characterization of a thermostable α-amylase from Nocardiopsis sp. endophyte of yam bean. Bioresour Technol 76:137–141. doi:10.1016/S0960-8524(00)00089-4 11131797

[40] Yeoman CJ, Han Y, Dodd D, Schroeder CM, Mackie RI, Cann IKO. 2010. Thermostable enzymes as biocatalysts in the biofuel industry. Adv Appl Microbiol 70:1–55. doi:10.1016/S0065-2164(10)70001-0 20359453 PMC4561533

[41] Stetter KO. 2006. History of discovery of the first hyperthermophiles. Extremophiles 10:357–362. doi:10.1007/s00792-006-0012-7 16941067

[42] Heuer VB, Inagaki F, Morono Y, Kubo Y, Spivack AJ, Viehweger B, Treude T, Beulig F, Schubotz F, Tonai S, et al.. 2020. Temperature limits to deep subseafloor life in the Nankai Trough subduction zone. Science 370:1230–1234. doi:10.1126/science.abd7934 33273103

[43] Kashefi K, Lovley DR. 2003. Extending the upper temperature limit for life. Science 301:934. doi:10.1126/science.1086823 12920290

[44] Majumdar S, Zhao P, Pfister NT, Compton M, Olson S, Glover CVC, Wells L, Graveley BR, Terns RM, Terns MP. 2015. Three CRISPR-Cas immune effector complexes coexist in Pyrococcus furiosus. RNA 21:1147–1158. doi:10.1261/rna.049130.114 25904135 PMC4436667

[45] Norais C, Moisan A, Gaspin C, Clouet-d’Orval B. 2013. Diversity of CRISPR systems in the euryarchaeal Pyrococcales. RNA Biol 10:659–670. doi:10.4161/rna.23927 23422322 PMC3737323

[46] Makarova KS, Haft DH, Barrangou R, Brouns SJJ, Charpentier E, Horvath P, Moineau S, Mojica FJM, Wolf YI, Yakunin AF, van der Oost J, Koonin EV. 2011. Evolution and classification of the CRISPR–Cas systems. Nat Rev Microbiol 9:467–477. doi:10.1038/nrmicro2577 21552286 PMC3380444

[47] Elmore JR, Yokooji Y, Sato T, Olson S, Glover CVC 3rd, Graveley BR, Atomi H, Terns RM, Terns MP. 2013. Programmable plasmid interference by the CRISPR-Cas system in Thermococcus kodakarensis. RNA Biol 10:828–840. doi:10.4161/rna.24084 23535213 PMC3737340

[48] Chylinski K, Makarova KS, Charpentier E, Koonin EV. 2014. Classification and evolution of type II CRISPR-Cas systems. Nucleic Acids Res 42:6091–6105. doi:10.1093/nar/gku241 24728998 PMC4041416

[49] Atomi H, Fukui T, Kanai T, Morikawa M, Imanaka T. 2004. Description of Thermococcus kodakaraensis sp. nov., a well studied hyperthermophilic archaeon previously reported as Pyrococcus sp. KOD1. Archaea 1:263–267. doi:10.1155/2004/204953 15810436 PMC2685570

[50] Takai K, Sugai A, Itoh T, Horikoshi K. 2000. Palaeococcus ferrophilus gen. nov., sp. nov., a barophilic, hyperthermophilic archaeon from a deep-sea hydrothermal vent chimney. Int J Syst Evol Microbiol 50:489–500. doi:10.1099/00207713-50-2-489 10758851

[51] Jolivet E, L’Haridon S, Corre E, Forterre P, Prieur D. 2003. Thermococcus gammatolerans sp. nov., a hyperthermophilic archaeon from a deep-sea hydrothermal vent that resists ionizing radiation. Int J Syst Evol Microbiol 53:847–851. doi:10.1099/ijs.0.02503-0 12807211

[52] Marteinsson VT, Birrien J-L, Reysenbach A-L, Vernet M, Marie D, Gambacorta A, Messner P, Sleytr UB, Prieur D. 1999. Thermococcus barophilus sp. nov., a new barophilic and hyperthermophilic archaeon isolated under high hydrostatic pressure from a deep-sea hydrothermal vent. Int J Syst Evol Microbiol 49:351–359. doi:10.1099/00207713-49-2-351 10319455

[53] Bae S-S, Kim Y-J, Yang S-H, Lim J-K, Jeon J-H, Lee H-S, Kang S-G, Kim S-J, Lee J-H. 2006. Thermococcus onnurineus sp. nov., a hyperthermophilic archaeon isolated from a deep-sea hydrothermal vent area at the PACMANUS field. J Microbiol Biotechnol 16:1826–1831.

[54] Gorlas A, Croce O, Oberto J, Gauliard E, Forterre P, Marguet E. 2014. Thermococcus nautili sp. nov., a hyperthermophilic archaeon isolated from a hydrothermal deep-sea vent. Int J Syst Evol Microbiol 64:1802–1810. doi:10.1099/ijs.0.060376-0 24556637

[55] Miroshnichenko ML, Hippe H, Stackebrandt E, Kostrikina NA, Chernyh NA, Jeanthon C, Nazina TN, Belyaev SS, Bonch-Osmolovskaya EA. 2001. Isolation and characterization of Thermococcus sibiricus sp. nov. from a Western Siberia high-temperature oil reservoir. Extremophiles 5:85–91. doi:10.1007/s007920100175 11354459

[56] Neuner A, Jannasch HW, Belkin S, Stetter KO. 1990. Thermococcus litoralis sp. nov.: a new species of extremely thermophilic marine archaebacteria. Arch Microbiol 153:205–207. doi:10.1007/BF00247822

[57] Huber R, Stöhr J, Hohenhaus S, Rachel R, Burggraf S, Jannasch HW, Stetter KO. 1995. Thermococcus chitonophagus sp. nov., a novel, chitin-degrading, hyperthermophilic archaeum from a deep-sea hydrothermal vent environment. Arch Microbiol 164:255–264. doi:10.1007/BF02529959

[58] Keller M, Braun FJ, Dirmeier R, Hafenbradl D, Burggraf S, Rachel R, Stetter KO. 1995. Thermococcus alcaliphilus sp. nov., a new hyperthermophilic archaeum growing on polysulfide at alkaline pH. Arch Microbiol 164:390–395. doi:10.1007/BF02529736 8588740

[59] Dirmeier R, Keller M, Hafenbradl D, Braun FJ, Rachel R, Burggraf S, Stetter KO. 1998. Thermococcus acidaminovorans sp. nov., a new hyperthermophilic alkalophilic archaeon growing on amino acids. Extremophiles 2:109–114. doi:10.1007/s007920050049 9672685

[60] Morikawa M, Izawa Y, Rashid N, Hoaki T, Imanaka T. 1994. Purification and characterization of a thermostable thiol protease from a newly isolated hyperthermophilic Pyrococcus sp. Appl Environ Microbiol 60:4559–4566. doi:10.1128/aem.60.12.4559-4566.1994 7811092 PMC202019

[61] Kawarabayasi Y. 1998. Complete sequence and gene organization of the genome of a hyper-thermophilic archaebacterium, Pyrococcus horikoshii OT3. DNA Res 5:55–76. doi:10.1093/dnares/5.2.55 9679194

[62] Fukui T, Atomi H, Kanai T, Matsumi R, Fujiwara S, Imanaka T. 2005. Complete genome sequence of the hyperthermophilic archaeon Thermococcus kodakaraensis KOD1 and comparison with Pyrococcus genomes. Genome Res 15:352–363. doi:10.1101/gr.3003105 15710748 PMC551561

[63] Lee HS, Kang SG, Bae SS, Lim JK, Cho Y, Kim YJ, Jeon JH, Cha S-S, Kwon KK, Kim H-T, Park C-J, Lee H-W, Kim SI, Chun J, Colwell RR, Kim S-J, Lee J-H. 2008. The complete genome sequence of Thermococcus onnurineus NA1 reveals a mixed heterotrophic and carboxydotrophic metabolism. J Bacteriol 190:7491–7499. doi:10.1128/JB.00746-08 18790866 PMC2576655

[64] Cohen GN, Barbe V, Flament D, Galperin M, Heilig R, Lecompte O, Poch O, Prieur D, Quérellou J, Ripp R, Thierry J-C, Van der Oost J, Weissenbach J, Zivanovic Y, Forterre P. 2003. An integrated analysis of the genome of the hyperthermophilic archaeon Pyrococcus abyssi. Mol Microbiol 47:1495–1512. doi:10.1046/j.1365-2958.2003.03381.x 12622808

[65] Robb FT, Maeder DL, Brown JR, DiRuggiero J, Stump MD, Yeh MK, Weiss RB, Dunn DM. 2001. Genomic sequence of hyperthermophile, *Pyrococcus furiosus*: implications for physiology and enzymology, p 134–157. In Methods in enzymology. Academic Press. 10.1016/s0076-6879(01)30372-5 11210495

[66] Zivanovic Y, Armengaud J, Lagorce A, Leplat C, Guérin P, Dutertre M, Anthouard V, Forterre P, Wincker P, Confalonieri F. 2009. Genome analysis and genome-wide proteomics of Thermococcus gammatolerans, the most radioresistant organism known amongst the Archaea. Genome Biol 10:R70. doi:10.1186/gb-2009-10-6-r70 19558674 PMC2718504

[67] Sato T, Fukui T, Atomi H, Imanaka T. 2003. Targeted gene disruption by homologous recombination in the hyperthermophilic archaeon Thermococcus kodakaraensis KOD1. J Bacteriol 185:210–220. doi:10.1128/JB.185.1.210-220.2003 12486058 PMC141832

[68] Sato T, Fukui T, Atomi H, Imanaka T. 2005. Improved and versatile transformation system allowing multiple genetic manipulations of the hyperthermophilic archaeon Thermococcus kodakaraensis. Appl Environ Microbiol 71:3889–3899. doi:10.1128/AEM.71.7.3889-3899.2005 16000802 PMC1169065

[69] Lipscomb GL, Stirrett K, Schut GJ, Yang F, Jenney FE, Scott RA, Adams MWW, Westpheling J. 2011. Natural competence in the hyperthermophilic archaeon Pyrococcus furiosus facilitates genetic manipulation: construction of markerless deletions of genes encoding the two cytoplasmic hydrogenases. Appl Environ Microbiol 77:2232–2238. doi:10.1128/AEM.02624-10 21317259 PMC3067412

[70] Thiel A, Michoud G, Moalic Y, Flament D, Jebbar M. 2014. Genetic manipulations of the hyperthermophilic piezophilic archaeon Thermococcus barophilus. Appl Environ Microbiol 80:2299–2306. doi:10.1128/AEM.00084-14 24487541 PMC3993132

[71] Kim YJ, Lee HS, Kim ES, Bae SS, Lim JK, Matsumi R, Lebedinsky AV, Sokolova TG, Kozhevnikova DA, Cha S-S, Kim S-J, Kwon KK, Imanaka T, Atomi H, Bonch-Osmolovskaya EA, Lee J-H, Kang SG. 2010. Formate-driven growth coupled with H_2_ production. Nature 467:352–355. doi:10.1038/nature09375 20844539

[72] Lucas S, Toffin L, Zivanovic Y, Charlier D, Moussard H, Forterre P, Prieur D, Erauso G. 2002. Construction of a shuttle vector for, and spheroplast transformation of, the hyperthermophilic archaeon Pyrococcus abyssi. Appl Environ Microbiol 68:5528–5536. doi:10.1128/AEM.68.11.5528-5536.2002 12406746 PMC129897

[73] Waege I, Schmid G, Thumann S, Thomm M, Hausner W. 2010. Shuttle vector-based transformation system for Pyrococcus furiosus. Appl Environ Microbiol 76:3308–3313. doi:10.1128/AEM.01951-09 20363792 PMC2869139

[74] Ikeda Y, Okada Y, Sato A, Kanai T, Tomita M, Atomi H, Kanai A. 2017. An archaeal RNA binding protein, FAU-1, is a novel ribonuclease related to rRNA stability in Pyrococcus and Thermococcus. Sci Rep 7:12674. doi:10.1038/s41598-017-13062-3 28978920 PMC5627344

[75] Liman GLS, Garcia AA, Fluke KA, Anderson HR, Davidson SC, Welander PV, Santangelo TJ. 2024. Tetraether archaeal lipids promote long-term survival in extreme conditions. Mol Microbiol 121:882–894. doi:10.1111/mmi.15240 38372181 PMC11096074

[76] Shimosaka T, Makarova KS, Koonin EV, Atomi H. 2019. Identification of dephospho-coenzyme a (dephospho-CoA) kinase in Thermococcus kodakarensis and elucidation of the entire CoA biosynthesis pathway in archaea. mBio 10:e01146-19. doi:10.1128/mBio.01146-19 31337720 PMC6650551

[77] Haja DK, Adams MWW. 2021. pH homeostasis and sodium ion pumping by multiple resistance and pH antiporters in Pyrococcus furiosus. Front Microbiol 12:712104. doi:10.3389/fmicb.2021.712104 34484150 PMC8415708

[78] Čuboňová L, Katano M, Kanai T, Atomi H, Reeve JN, Santangelo TJ. 2012. An archaeal histone is required for transformation of Thermococcus kodakarensis. J Bacteriol 194:6864–6874. doi:10.1128/JB.01523-12 23065975 PMC3510624

[79] Tagashira K, Fukuda W, Matsubara M, Kanai T, Atomi H, Imanaka T. 2013. Genetic studies on the virus-like regions in the genome of hyperthermophilic archaeon, Thermococcus kodakarensis. Extremophiles 17:153–160. doi:10.1007/s00792-012-0504-6 23224520

[80] Hirata A, Kanai T, Santangelo TJ, Tajiri M, Manabe K, Reeve JN, Imanaka T, Murakami KS. 2008. Archaeal RNA polymerase subunits E and F are not required for transcription in vitro, but a Thermococcus kodakarensis mutant lacking subunit F is temperature-sensitive. Mol Microbiol 70:623–633. doi:10.1111/j.1365-2958.2008.06430.x 18786148 PMC3737576

[81] Walker JE, Luyties O, Santangelo TJ. 2017. Factor-dependent archaeal transcription termination. Proc Natl Acad Sci USA 114:E6767–E6773. doi:10.1073/pnas.1704028114 28760969 PMC5565431

[82] Hidese R, Nishikawa R, Gao L, Katano M, Imai T, Kato S, Kanai T, Atomi H, Imanaka T, Fujiwara S. 2014. Different roles of two transcription factor B proteins in the hyperthermophilic archaeon Thermococcus kodakarensis. Extremophiles 18:573–588. doi:10.1007/s00792-014-0638-9 24627188

[83] Nagata M, Ishino S, Yamagami T, Ogino H, Simons J-R, Kanai T, Atomi H, Ishino Y. 2017. The Cdc45/RecJ-like protein forms a complex with GINS and MCM, and is important for DNA replication in Thermococcus kodakarensis. Nucleic Acids Res 45:10693–10705. doi:10.1093/nar/gkx740 28977567 PMC5737688

[84] Caffrey PJ, Eckenroth BE, Burkhart BW, Zatopek KM, McClung CM, Santangelo TJ, Doublié S, Gardner AF. 2024. Thermococcus kodakarensis TK0353 is a novel AP lyase with a new fold. J Biol Chem 300:105503. doi:10.1016/j.jbc.2023.105503 38013090 PMC10731606

[85] Liman GLS, Lennon CW, Mandley JL, Galyon AM, Zatopek KM, Gardner AF, Santangelo TJ. 2024. Intein splicing efficiency and RadA levels can control the mode of archaeal DNA replication. Sci Adv 10:eadp4995. doi:10.1126/sciadv.adp4995 39292776 PMC11409957

[86] Fujiwara S, Aki R, Yoshida M, Higashibata H, Imanaka T, Fukuda W. 2008. Expression profiles and physiological roles of two types of molecular chaperonins from the hyperthermophilic archaeon Thermococcus kodakarensis. Appl Environ Microbiol 74:7306–7312. doi:10.1128/AEM.01245-08 18835998 PMC2592908

[87] Danno A, Fukuda W, Yoshida M, Aki R, Tanaka T, Kanai T, Imanaka T, Fujiwara S. 2008. Expression profiles and physiological roles of two types of prefoldins from the hyperthermophilic archaeon Thermococcus kodakaraensis. J Mol Biol 382:298–311. doi:10.1016/j.jmb.2008.07.032 18662698

[88] Scott KA, Williams SA, Santangelo TJ. 2021. *Thermococcus kodakarensis* provides a versatile hyperthermophilic archaeal platform for protein expression, p. 243–273. In Kelman, Z, O’Dell, WB (eds.), Methods in enzymology. Academic Press. 10.1016/bs.mie.2021.06.014 PMC8878339 34752288

[89] Zatopek KM, Burkhart BW, Morgan RD, Gehring AM, Scott KA, Santangelo TJ, Gardner AF. 2021. The hyperthermophilic restriction-modification systems of Thermococcus kodakarensis protect genome integrity. Front Microbiol 12:657356. doi:10.3389/fmicb.2021.657356 34093470 PMC8172983

[90] Catchpole RJ, Barbe V, Magdelenat G, Marguet E, Terns M, Oberto J, Forterre P, Da Cunha V. 2023. A self-transmissible plasmid from a hyperthermophile that facilitates genetic modification of diverse Archaea. Nat Microbiol 8:1339–1347. doi:10.1038/s41564-023-01387-x 37277532 PMC10788138

[91] Mai X, Adams MW. 1994. Indolepyruvate ferredoxin oxidoreductase from the hyperthermophilic archaeon Pyrococcus furiosus. A new enzyme involved in peptide fermentation. J Biol Chem 269:16726–16732. doi:10.1016/S0021-9258(19)89451-6 8206994

[92] Heider J, Mai X, Adams MW. 1996. Characterization of 2-ketoisovalerate ferredoxin oxidoreductase, a new and reversible coenzyme A-dependent enzyme involved in peptide fermentation by hyperthermophilic archaea. J Bacteriol 178:780–787. doi:10.1128/jb.178.3.780-787.1996 8550513 PMC177725

[93] Smith ET, Blamey JM, Adams MWW. 1994. Pyruvate ferredoxin oxidoreductases of the hyperthermophilic archaeon, Pyrococcus furiosus, and the hyperthermophilic bacterium, Thermotoga maritima, have different catalytic mechanisms. Biochemistry 33:1008–1016. doi:10.1021/bi00170a020 8305427

[94] Glasemacher J, Bock AK, Schmid R, Schønheit P. 1997. Purification and properties of acetyl-CoA synthetase (ADP-forming), an archaeal enzyme of acetate formation and ATP synthesis, from the hyperthermophile Pyrococcus furiosus. Eur J Biochem 244:561–567. doi:10.1111/j.1432-1033.1997.00561.x 9119024

[95] Mai X, Adams MW. 1996. Purification and characterization of two reversible and ADP-dependent acetyl coenzyme A synthetases from the hyperthermophilic archaeon Pyrococcus furiosus. J Bacteriol 178:5897–5903. doi:10.1128/jb.178.20.5897-5903.1996 8830684 PMC178444

[96] Awano T, Wilming A, Tomita H, Yokooji Y, Fukui T, Imanaka T, Atomi H. 2014. Characterization of two members among the five ADP-forming acyl coenzyme A (Acyl-CoA) synthetases reveals the presence of a 2-(Imidazol-4-yl)acetyl-CoA synthetase in Thermococcus kodakarensis. J Bacteriol 196:140–147. doi:10.1128/JB.00877-13 24163338 PMC3911136

[97] Mukund S, Adams MWW. 1995. Glyceraldehyde-3-phosphate ferredoxin oxidoreductase, a novel tungsten-containing enzyme with a potential glycolytic role in the hyperthermophilic archaeon Pyrococcus furiosus. J Biol Chem 270:8389–8392. doi:10.1074/jbc.270.15.8389 7721730

[98] Kengen SW, de Bok FA, van Loo ND, Dijkema C, Stams AJ, de Vos WM. 1994. Evidence for the operation of a novel Embden-Meyerhof pathway that involves ADP-dependent kinases during sugar fermentation by Pyrococcus furiosus. J Biol Chem 269:17537–17541. doi:10.1016/S0021-9258(17)32474-2 8021261

[99] Sakuraba H, Ohshima T. 2002. Novel energy metabolism in anaerobic hyperthermophilic archaea: a modified Embden-Meyerhof pathway. J Biosci Bioeng 93:441–448. doi:10.1016/s1389-1723(02)80090-1 16233230

[100] Siebers B, Schönheit P. 2005. Unusual pathways and enzymes of central carbohydrate metabolism in Archaea. Curr Opin Microbiol 8:695–705. doi:10.1016/j.mib.2005.10.014 16256419

[101] Tuininga JE, Verhees CH, van der Oost J, Kengen SWM, Stams AJM, de Vos WM. 1999. Molecular and biochemical characterization of the ADP-dependent phosphofructokinase from the hyperthermophilic archaeon Pyrococcus furiosus. J Biol Chem 274:21023–21028. doi:10.1074/jbc.274.30.21023 10409652

[102] Wu C-H, Schut GJ, Poole FL, Haja DK, Adams MWW. 2018. Characterization of membrane-bound sulfane reductase: a missing link in the evolution of modern day respiratory complexes. J Biol Chem 293:16687–16696. doi:10.1074/jbc.RA118.005092 30181217 PMC6204914

[103] Xiao X, Schut GJ, Feng X, McTernan PM, Haja DK, Lanzilotta WN, Adams MWW, Li H. 2025. Structural insights into the biotechnologically relevant reversible NADPH-oxidizing NiFe-hydrogenase from P. furiosus. Structure 33:1470–1483. doi:10.1016/j.str.2025.05.017 40570843

[104] Burkhart BW, Febvre HP, Santangelo TJ. 2019. Distinct physiological roles of the three ferredoxins encoded in the hyperthermophilic archaeon Thermococcus kodakarensis. mBio 10:e02807-18. doi:10.1128/mBio.02807-18 30837343 PMC6401487

[105] Lipscomb GL, Schut GJ, Thorgersen MP, Nixon WJ, Kelly RM, Adams MWW. 2014. Engineering hydrogen gas production from formate in a hyperthermophile by heterologous production of an 18-subunit membrane-bound complex. J Biol Chem 289:2873–2879. doi:10.1074/jbc.M113.530725 24318960 PMC3908419

[106] Wu C-H, McTernan PM, Walter ME, Adams MWW. 2015. Production and application of a soluble hydrogenase from Pyrococcus furiosus. Archaea 2015:912582. doi:10.1155/2015/912582 26543406 PMC4620386

[107] Lim JK, Mayer F, Kang SG, Müller V. 2014. Energy conservation by oxidation of formate to carbon dioxide and hydrogen via a sodium ion current in a hyperthermophilic archaeon. Proc Natl Acad Sci USA 111:11497–11502. doi:10.1073/pnas.1407056111 25049407 PMC4128143

[108] Schut GJ, Lipscomb GL, Nguyen DMN, Kelly RM, Adams MWW. 2016. Heterologous production of an energy-conserving carbon monoxide dehydrogenase complex in the hyperthermophile Pyrococcus furiosus. Front Microbiol 7:29. doi:10.3389/fmicb.2016.00029 26858706 PMC4731540

[109] Lee SH, Kim M-S, Lee J-H, Kim TW, Bae SS, Lee S-M, Jung HC, Yang T-J, Choi AR, Cho Y-J, Lee J-H, Kwon KK, Lee HS, Kang SG. 2016. Adaptive engineering of a hyperthermophilic archaeon on CO and discovering the underlying mechanism by multi-omics analysis. Sci Rep 6:22896. doi:10.1038/srep22896 26975345 PMC4791640

[110] Ezaki S, Maeda N, Kishimoto T, Atomi H, Imanaka T. 1999. Presence of a structurally novel type ribulose-bisphosphate carboxylase/oxygenase in the hyperthermophilic archaeon, Pyrococcus kodakaraensis KOD1. J Biol Chem 274:5078–5082. doi:10.1074/jbc.274.8.5078 9988755

[111] Sato T, Atomi H, Imanaka T. 2007. Archaeal type III RuBisCOs function in a pathway for AMP metabolism. Science 315:1003–1006. doi:10.1126/science.1135999 17303759

[112] Aono R, Sato T, Imanaka T, Atomi H. 2015. A pentose bisphosphate pathway for nucleoside degradation in Archaea. Nat Chem Biol 11:355–360. doi:10.1038/nchembio.1786 25822915

[113] Horiuchi A, Aslam M, Kanai T, Atomi H. 2016. A structurally novel chitinase from the chitin-degrading hyperthermophilic archaeon Thermococcus chitonophagus. Appl Environ Microbiol 82:3554–3562. doi:10.1128/AEM.00319-16 27060120 PMC4959179

[114] Chohan SM, Rashid N. 2013. TK1656, a thermostable l-asparaginase from Thermococcus kodakaraensis, exhibiting highest ever reported enzyme activity. J Biosci Bioeng 116:438–443. doi:10.1016/j.jbiosc.2013.04.005 23648103

[115] Mathew LG, Haja DK, Pritchett C, McCormick W, Zeineddine R, Fontenot LS, Rivera ME, Glushka J, Adams MWW, Lanzilotta WN. 2022. An unprecedented function for a tungsten-containing oxidoreductase. J Biol Inorg Chem 27:747–758. doi:10.1007/s00775-022-01965-0 36269456 PMC10293930

[116] Johnson MK, Rees DC, Adams MWW. 1996. Tungstoenzymes. Chem Rev 96:2817–2840. doi:10.1021/cr950063d 11848842

[117] Chan MK, Mukund S, Kletzin A, Adams MWW, Rees DC. 1995. Structure of a hyperthermophilic tungstopterin enzyme, aldehyde ferredoxin oxidoreductase. Science 267:1463–1469. doi:10.1126/science.7878465 7878465

[118] Lee HS, Kim YJ, Bae SS, Jeon JH, Lim JK, Jeong BC, Kang SG, Lee J-H. 2006. Cloning, expression, and characterization of aminopeptidase P from the hyperthermophilic archaeon Thermococcus sp. strain NA1. Appl Environ Microbiol 72:1886–1890. doi:10.1128/AEM.72.3.1886-1890.2006 16517635 PMC1393192

[119] Haja DK, Wu C-H, Ponomarenko O, Poole FL, George GN, Adams MWW. 2020. Improving arsenic tolerance of Pyrococcus furiosus by heterologous expression of a respiratory arsenate reductase. Appl Environ Microbiol 86:e01728-20. doi:10.1128/AEM.01728-20 32859593 PMC7580539

[120] Cvetkovic A, Menon AL, Thorgersen MP, Scott JW, Poole II FL, Jenney Jr FE, Lancaster WA, Praissman JL, Shanmukh S, Vaccaro BJ, Trauger SA, Kalisiak E, Apon JV, Siuzdak G, Yannone SM, Tainer JA, Adams MWW. 2010. Microbial metalloproteomes are largely uncharacterized. Nature 466:779–782. doi:10.1038/nature09265 20639861

[121] Day MW, Hsu BT, Joshua‐Tor L, Park J, Zhou ZH, Adams MWW, Rees DC. 1992. X-ray crystal structures of the oxidized and reduced forms of the rubredoxin from the marine hyperthermophilic archaebacterium Pyrococcus furiosus. Protein Sci 1:1494–1507. doi:10.1002/pro.5560011111 1303768 PMC2142115

[122] Martínez-Carranza M, Vialle L, Madru C, Cordier F, Tekpinar AD, Haouz A, Legrand P, Le Meur RA, England P, Dulermo R, Guijarro JI, Henneke G, Sauguet L. 2024. Communication between DNA polymerases and Replication Protein A within the archaeal replisome. Nat Commun 15:10926. doi:10.1038/s41467-024-55365-w 39738083 PMC11686378

[123] Tarău D, Grünberger F, Pilsl M, Reichelt R, Heiß F, König S, Urlaub H, Hausner W, Engel C, Grohmann D. 2024. Structural basis of archaeal RNA polymerase transcription elongation and Spt4/5 recruitment. Nucleic Acids Res 52:6017–6035. doi:10.1093/nar/gkae282 38709902 PMC11162788

[124] You L, Wang C, Molodtsov V, Kuznedelov K, Miao X, Wenck BR, Ulisse P, Sanders TJ, Marshall CJ, Firlar E, Kaelber JT, Santangelo TJ, Ebright RH. 2024. Structural basis of archaeal FttA-dependent transcription termination. Nature 635:229–236. doi:10.1038/s41586-024-07979-9 39322680 PMC11616081

[125] Vonck J, Pisa KY, Morgner N, Brutschy B, Müller V. 2009. Three-dimensional structure of A1A0 ATP synthase from the hyperthermophilic archaeon Pyrococcus furiosus by electron microscopy. J Biol Chem 284:10110–10119. doi:10.1074/jbc.M808498200 19203996 PMC2665065

[126] Rees DC, Robertson AD. 2001. Some thermodynamic implications for the thermostability of proteins. Protein Sci 10:1187–1194. doi:10.1110/ps.180101 11369857 PMC2374017

[127] Maeda N, Kanai T, Atomi H, Imanaka T. 2002. The unique pentagonal structure of an archaeal Rubisco is essential for its high thermostability. J Biol Chem 277:31656–31662. doi:10.1074/jbc.M203117200 12070156

[128] Yu H, Wu C-H, Schut GJ, Haja DK, Zhao G, Peters JW, Adams MWW, Li H. 2018. Structure of an ancient respiratory system. Cell 173:1636–1649. doi:10.1016/j.cell.2018.03.071 29754813 PMC6003862

[129] Siglioccolo A, Paiardini A, Piscitelli M, Pascarella S. 2011. Structural adaptation of extreme halophilic proteins through decrease of conserved hydrophobic contact surface. BMC Struct Biol 11:50. doi:10.1186/1472-6807-11-50 22192175 PMC3293032

[130] Stevens KM, Hocher A, Warnecke T. 2022. Deep conservation of histone variants in Thermococcales archaea. Genome Biol Evol 14:evab274. doi:10.1093/gbe/evab274 34894218 PMC8775648

[131] Tapias A, Leplat C, Confalonieri F. 2009. Recovery of ionizing-radiation damage after high doses of gamma ray in the hyperthermophilic archaeon Thermococcus gammatolerans. Extremophiles 13:333–343. doi:10.1007/s00792-008-0221-3 19137239

[132] DiRuggiero J, Santangelo N, Nackerdien Z, Ravel J, Robb FT. 1997. Repair of extensive ionizing-radiation DNA damage at 95 degrees C in the hyperthermophilic archaeon Pyrococcus furiosus. J Bacteriol 179:4643–4645. doi:10.1128/jb.179.14.4643-4645.1997 9226280 PMC179306

[133] Jolivet E, Corre E, L’Haridon S, Forterre P, Prieur D. 2004. Thermococcus marinus sp. nov. and Thermococcus radiotolerans sp. nov., two hyperthermophilic archaea from deep-sea hydrothermal vents that resist ionizing radiation. Extremophiles 8:219–227. doi:10.1007/s00792-004-0380-9 14991422

[134] Marshall CJ, Santangelo TJ. 2020. Archaeal DNA repair mechanisms. Biomolecules 10:1472. doi:10.3390/biom10111472 33113933 PMC7690668

[135] Kelman Z, White MF. 2005. Archaeal DNA replication and repair. Curr Opin Microbiol 8:669–676. doi:10.1016/j.mib.2005.10.001 16242991

[136] Gehring AM, Zatopek KM, Burkhart BW, Potapov V, Santangelo TJ, Gardner AF. 2020. Biochemical reconstitution and genetic characterization of the major oxidative damage base excision DNA repair pathway in Thermococcus kodakarensis. DNA Repair (Amst) 86:102767. doi:10.1016/j.dnarep.2019.102767 31841800 PMC8061334

[137] Forterre P. 2002. A hot story from comparative genomics: reverse gyrase is the only hyperthermophile-specific protein. Trends Genet 18:236–237. doi:10.1016/s0168-9525(02)02650-1 12047940

[138] Bouthier de la Tour C, Portemer C, Nadal M, Stetter KO, Forterre P, Duguet M. 1990. Reverse gyrase, a hallmark of the hyperthermophilic archaebacteria. J Bacteriol 172:6803–6808. doi:10.1128/jb.172.12.6803-6808.1990 2174859 PMC210796

[139] Guipaud O, Marguet E, Noll KM, de la Tour CB, Forterre P. 1997. Both DNA gyrase and reverse gyrase are present in the hyperthermophilic bacterium Thermotoga maritima. Proc Natl Acad Sci USA 94:10606–10611. doi:10.1073/pnas.94.20.10606 9380682 PMC23419

[140] López-García P, Forterre P, van der Oost J, Erauso G. 2000. Plasmid pGS5 from the hyperthermophilic archaeon Archaeoglobus profundus is negatively supercoiled. J Bacteriol 182:4998–5000. doi:10.1128/JB.182.17.4998-5000.2000 10940047 PMC111383

[141] Villain P, da Cunha V, Villain E, Forterre P, Oberto J, Catchpole R, Basta T. 2021. The hyperthermophilic archaeon Thermococcus kodakarensis is resistant to pervasive negative supercoiling activity of DNA gyrase. Nucleic Acids Res 49:12332–12347. doi:10.1093/nar/gkab869 34755863 PMC8643681

[142] Atomi H, Matsumi R, Imanaka T. 2004. Reverse gyrase is not a prerequisite for hyperthermophilic life. J Bacteriol 186:4829–4833. doi:10.1128/JB.186.14.4829-4833.2004 15231817 PMC438624

[143] Mills KV, Johnson MA, Perler FB. 2014. Protein splicing: how inteins escape from precursor proteins. J Biol Chem 289:14498–14505. doi:10.1074/jbc.R113.540310 24695729 PMC4031507

[144] Novikova O, Topilina N, Belfort M. 2014. Enigmatic distribution, evolution, and function of inteins. J Biol Chem 289:14490–14497. doi:10.1074/jbc.R114.548255 24695741 PMC4031506

[145] Lennon CW, Stanger M, Banavali NK, Belfort M. 2018. Conditional protein splicing switch in hyperthermophiles through an intein-extein partnership. mBio 9:e02304-17. doi:10.1128/mBio.02304-17 29382734 PMC5790916

[146] Perler FB, Comb DG, Jack WE, Moran LS, Qiang B, Kucera RB, Benner J, Slatko BE, Nwankwo DO, Hempstead SK. 1992. Intervening sequences in an Archaea DNA polymerase gene. Proc Natl Acad Sci USA 89:5577–5581. doi:10.1073/pnas.89.12.5577 1608969 PMC49335

[147] Southworth MW, Kong H, Kucera RB, Ware J, Jannasch HW, Perler FB. 1996. Cloning of thermostable DNA polymerases from hyperthermophilic marine Archaea with emphasis on Thermococcus sp. 9 degrees N-7 and mutations affecting 3’-5’ exonuclease activity. Proc Natl Acad Sci USA 93:5281–5285. doi:10.1073/pnas.93.11.5281 8643567 PMC39236

[148] Nanda A, Nasker SS, Mehra A, Panda S, Nayak S. 2020. Inteins in science: evolution to application. Microorganisms 8:2004. doi:10.3390/microorganisms8122004 33339089 PMC7765530

[149] Wood DW, Camarero JA. 2014. Intein applications: from protein purification and labeling to metabolic control methods. J Biol Chem 289:14512–14519. doi:10.1074/jbc.R114.552653 24700459 PMC4031509

[150] Ishino Y, Komori K, Cann IKO, Koga Y. 1998. A novel DNA polymerase family found in Archaea. J Bacteriol 180:2232–2236. doi:10.1128/JB.180.8.2232-2236.1998 9555910 PMC107154

[151] Čuboňová L, Richardson T, Burkhart BW, Kelman Z, Connolly BA, Reeve JN, Santangelo TJ. 2013. Archaeal DNA polymerase D but not DNA polymerase B is required for genome replication in Thermococcus kodakarensis. J Bacteriol 195:2322–2328. doi:10.1128/JB.02037-12 23504010 PMC3650531

[152] Sauguet L, Raia P, Henneke G, Delarue M. 2016. Shared active site architecture between archaeal PolD and multi-subunit RNA polymerases revealed by X-ray crystallography. Nat Commun 7:12227. doi:10.1038/ncomms12227 27548043 PMC4996933

[153] Komori K, Sakae S, Shinagawa H, Morikawa K, Ishino Y. 1999. A Holliday junction resolvase from Pyrococcus furiosus: functional similarity to Escherichia coli RuvC provides evidence for conserved mechanism of homologous recombination in Bacteria, Eukarya, and Archaea. Proc Natl Acad Sci USA 96:8873–8878. doi:10.1073/pnas.96.16.8873 10430863 PMC17700

[154] Komori K, Fujikane R, Shinagawa H, Ishino Y. 2002. Novel endonuclease in Archaea cleaving DNA with various branched structure. Genes Genet Syst 77:227–241. doi:10.1266/ggs.77.227 12419895

[155] Komori K, Hidaka M, Horiuchi T, Fujikane R, Shinagawa H, Ishino Y. 2004. Cooperation of the N-terminal helicase and C-terminal endonuclease activities of Archaeal Hef protein in processing stalled replication forks. J Biol Chem 279:53175–53185. doi:10.1074/jbc.M409243200 15485882

[156] Meetei AR, Medhurst AL, Ling C, Xue Y, Singh TR, Bier P, Steltenpool J, Stone S, Dokal I, Mathew CG, Hoatlin M, Joenje H, de Winter JP, Wang W. 2005. A human ortholog of archaeal DNA repair protein Hef is defective in Fanconi anemia complementation group M. Nat Genet 37:958–963. doi:10.1038/ng1626 16116422 PMC2704909

[157] Mosedale G, Niedzwiedz W, Alpi A, Perrina F, Pereira-Leal JB, Johnson M, Langevin F, Pace P, Patel KJ. 2005. The vertebrate Hef ortholog is a component of the Fanconi anemia tumor-suppressor pathway. Nat Struct Mol Biol 12:763–771. doi:10.1038/nsmb981 16116434

[158] Li Z, Santangelo TJ, Cuboňová L, Reeve JN, Kelman Z. 2010. Affinity purification of an archaeal DNA replication protein network. mBio 1:e00221-10. doi:10.1128/mBio.00221-10 20978540 PMC2962436

[159] Gehring AM, Astling DP, Matsumi R, Burkhart BW, Kelman Z, Reeve JN, Jones KL, Santangelo TJ. 2017. Genome replication in Thermococcus kodakarensis independent of Cdc6 and an origin of replication. Front Microbiol 8:2084. doi:10.3389/fmicb.2017.02084 29163389 PMC5663688

[160] Mc Teer L, Moalic Y, Cueff-Gauchard V, Catchpole R, Hogrel G, Lu Y, Laurent S, Hemon M, Aubé J, Leroy E, Roussel E, Oberto J, Flament D, Dulermo R. 2024. Cooperation between two modes for DNA replication initiation in the archaeon Thermococcus barophilus. mBio 15:e0320023. doi:10.1128/mbio.03200-23 38421162 PMC11005403

[161] Greenough L, Kelman Z, Gardner AF. 2015. The roles of family B and D DNA polymerases in Thermococcus species 9°N Okazaki fragment maturation. J Biol Chem 290:12514–12522. doi:10.1074/jbc.M115.638130 25814667 PMC4432273

[162] Matsunaga F, Forterre P, Ishino Y, Myllykallio H. 2001. In vivo interactions of archaeal Cdc6/Orc1 and minichromosome maintenance proteins with the replication origin. Proc Natl Acad Sci USA 98:11152–11157. doi:10.1073/pnas.191387498 11562464 PMC58699

[163] Myllykallio H, Lopez P, López-García P, Heilig R, Saurin W, Zivanovic Y, Philippe H, Forterre P. 2000. Bacterial mode of replication with eukaryotic-like machinery in a hyperthermophilic archaeon. Science 288:2212–2215. doi:10.1126/science.288.5474.2212 10864870

[164] Spaans SK, van der Oost J, Kengen SWM. 2015. The chromosome copy number of the hyperthermophilic archaeon Thermococcus kodakarensis KOD1. Extremophiles 19:741–750. doi:10.1007/s00792-015-0750-5 25952670 PMC4502288

[165] Werner F, Grohmann D. 2011. Evolution of multisubunit RNA polymerases in the three domains of life. Nat Rev Microbiol 9:85–98. doi:10.1038/nrmicro2507 21233849

[166] Hethke C, Geerling AC, Hausner W, de Vos WM, Thomm M. 1996. A cell-free transcription system for the hyperthermophilic archaeon Pyrococcus furiosus. Nucleic Acids Res 24:2369–2376. doi:10.1093/nar/24.12.2369 8710509 PMC145958

[167] Blombach F, Smollett KL, Grohmann D, Werner F. 2016. Molecular mechanisms of transcription initiation—structure, function, and evolution of TFE/TFIIE-like factors and open complex formation. J Mol Biol 428:2592–2606. doi:10.1016/j.jmb.2016.04.016 27107643 PMC7616663

[168] Santangelo TJ, Cubonová L, James CL, Reeve JN. 2007. TFB1 or TFB2 is sufficient for Thermococcus kodakaraensis viability and for basal transcription in vitro. J Mol Biol 367:344–357. doi:10.1016/j.jmb.2006.12.069 17275836 PMC1855253

[169] Micorescu M, Grünberg S, Franke A, Cramer P, Thomm M, Bartlett M. 2008. Archaeal transcription: function of an alternative transcription factor B from Pyrococcus furiosus. J Bacteriol 190:157–167. doi:10.1128/JB.01498-07 17965161 PMC2223750

[170] Hausner W, Wettach J, Hethke C, Thomm M. 1996. Two transcription factors related with the eucaryal transcription factors TATA-binding protein and transcription factor IIB direct promoter recognition by an archaeal RNA polymerase. J Biol Chem 271:30144–30148. doi:10.1074/jbc.271.47.30144 8939964

[171] Sanders TJ, Lammers M, Marshall CJ, Walker JE, Lynch ER, Santangelo TJ. 2019. TFS and Spt4/5 accelerate transcription through archaeal histone-based chromatin. Mol Microbiol 111:784–797. doi:10.1111/mmi.14191 30592095 PMC6417941

[172] Grünberg S, Bartlett MS, Naji S, Thomm M. 2007. Transcription factor E is a part of transcription elongation complexes. J Biol Chem 282:35482–35490. doi:10.1074/jbc.M707371200 17921145

[173] Wenck BR, Vickerman RL, Burkhart BW, Santangelo TJ. 2024. Archaeal histone-based chromatin structures regulate transcription elongation rates. Commun Biol 7:236. doi:10.1038/s42003-024-05928-w 38413771 PMC10899632

[174] Santangelo TJ, Cubonová L, Skinner KM, Reeve JN. 2009. Archaeal intrinsic transcription termination in vivo. J Bacteriol 191:7102–7108. doi:10.1128/JB.00982-09 19749050 PMC2772485

[175] Sanders TJ, Wenck BR, Selan JN, Barker MP, Trimmer SA, Walker JE, Santangelo TJ. 2020. FttA is a CPSF73 homologue that terminates transcription in Archaea. Nat Microbiol 5:545–553. doi:10.1038/s41564-020-0667-3 32094586 PMC7103508

[176] Phung DK, Rinaldi D, Langendijk-Genevaux PS, Quentin Y, Carpousis AJ, Clouet-d’Orval B. 2013. Archaeal β-CASP ribonucleases of the aCPSF1 family are orthologs of the eukaryal CPSF-73 factor. Nucleic Acids Res 41:1091–1103. doi:10.1093/nar/gks1237 23222134 PMC3553952

[177] Stöckl R, Nißl L, Reichelt R, Rachel R, Grohmann D, Grünberger F. 2023. The transcriptional regulator EarA and intergenic terminator sequences modulate archaellation in Pyrococcus furiosus. Front Microbiol 14:1241399. doi:10.3389/fmicb.2023.1241399 38029142 PMC10665913

[178] Lemmens L, Maklad HR, Bervoets I, Peeters E. 2019. Transcription regulators in Archaea: homologies and differences with bacterial regulators. J Mol Biol 431:4132–4146. doi:10.1016/j.jmb.2019.05.045 31195017

[179] Bell SD, Jackson SP. 2001. Mechanism and regulation of transcription in archaea. Curr Opin Microbiol 4:208–213. doi:10.1016/s1369-5274(00)00190-9 11282478

[180] Faure G, Ogurtsov AY, Shabalina SA, Koonin EV. 2016. Role of mRNA structure in the control of protein folding. Nucleic Acids Res 44:10898–10911. doi:10.1093/nar/gkw671 27466388 PMC5159526

[181] French SL, Santangelo TJ, Beyer AL, Reeve JN. 2007. Transcription and translation are coupled in Archaea. Mol Biol Evol 24:893–895. doi:10.1093/molbev/msm007 17237472

[182] Lepage E, Marguet E, Geslin C, Matte-Tailliez O, Zillig W, Forterre P, Tailliez P. 2004. Molecular diversity of new Thermococcales isolates from a single area of hydrothermal deep-sea vents as revealed by randomly amplified polymorphic DNA fingerprinting and 16S rRNA gene sequence analysis. Appl Environ Microbiol 70:1277–1286. doi:10.1128/AEM.70.3.1277-1286.2004 15006744 PMC368356

[183] Krupovic M, Gonnet M, Hania WB, Forterre P, Erauso G. 2013. Insights into dynamics of mobile genetic elements in hyperthermophilic environments from five new Thermococcus plasmids. PLoS One 8:e49044. doi:10.1371/journal.pone.0049044 23326305 PMC3543421

[184] Soler N, Marguet E, Verbavatz J-M, Forterre P. 2008. Virus-like vesicles and extracellular DNA produced by hyperthermophilic archaea of the order Thermococcales. Res Microbiol 159:390–399. doi:10.1016/j.resmic.2008.04.015 18625304

[185] Prieur D, Erauso G, Geslin C, Lucas S, Gaillard M, Bidault A, Mattenet A-C, Rouault K, Flament D, Forterre P, Le Romancer M. 2004. Genetic elements of Thermococcales. Biochem Soc Trans 32:184–187. doi:10.1042/bst0320184 15046568

[186] Soler N, Gaudin M, Marguet E, Forterre P. 2011. Plasmids, viruses and virus-like membrane vesicles from Thermococcales. Biochem Soc Trans 39:36–44. doi:10.1042/BST0390036 21265744

[187] Badel C, Erauso G, Gomez AL, Catchpole R, Gonnet M, Oberto J, Forterre P, Da Cunha V. 2019. The global distribution and evolutionary history of the pT26-2 archaeal plasmid family. Environ Microbiol 21:4685–4705. doi:10.1111/1462-2920.14800 31503394 PMC6972569

[188] Soler N, Justome A, Quevillon-Cheruel S, Lorieux F, Le Cam E, Marguet E, Forterre P. 2007. The rolling-circle plasmid pTN1 from the hyperthermophilic archaeon Thermococcus nautilus. Mol Microbiol 66:357–370. doi:10.1111/j.1365-2958.2007.05912.x 17784911

[189] Forterre P, Krupovic M, Raymann K, Soler N. 2014. Plasmids from Euryarchaeota. Microbiol Spectr 2. doi:10.1128/microbiolspec.PLAS-0027-2014 26104461

[190] Prieur D, Erauso G, Flament D, Gaillard M, Geslin C, Gonnet M, Romancer ML, Lucas S, Forterrre P. 2006. 11 deep-sea *Thermococcales* and their genetic elements: plasmids and viruses, p 253–278. In Methods in microbiology. Academic Press.

[191] Benbouzid-Rollet N, López-García P, Watrin L, Erauso G, Prieur D, Forterre P. 1997. Isolation of new plasmids from hyperthermophilic Archaea of the order Thermococcales. Res Microbiol 148:767–775. doi:10.1016/S0923-2508(97)82452-7 9765860

[192] Geslin C, Le Romancer M, Erauso G, Gaillard M, Perrot G, Prieur D. 2003. PAV1, the first virus-like particle isolated from a hyperthermophilic euryarchaeote, “Pyrococcus abyssi”. J Bacteriol 185:3888–3894. doi:10.1128/JB.185.13.3888-3894.2003 12813083 PMC161591

[193] Gorlas A, Koonin EV, Bienvenu N, Prieur D, Geslin C. 2012. TPV1, the first virus isolated from the hyperthermophilic genus Thermococcus. Environ Microbiol 14:503–516. doi:10.1111/j.1462-2920.2011.02662.x 22151304 PMC5935114

[194] Akita F, Chong KT, Tanaka H, Yamashita E, Miyazaki N, Nakaishi Y, Suzuki M, Namba K, Ono Y, Tsukihara T, Nakagawa A. 2007. The crystal structure of a virus-like particle from the hyperthermophilic archaeon Pyrococcus furiosus provides insight into the evolution of viruses. J Mol Biol 368:1469–1483. doi:10.1016/j.jmb.2007.02.075 17397865

[195] Portillo MC, Gonzalez JM. 2009. CRISPR elements in the Thermococcales: evidence for associated horizontal gene transfer in Pyrococcus furiosus. J Appl Genet 50:421–430. doi:10.1007/BF03195703 19875895

[196] Starostina NG, Marshburn S, Johnson LS, Eddy SR, Terns RM, Terns MP. 2004. Circular box C/D RNAs in Pyrococcus furiosus. Proc Natl Acad Sci USA 101:14097–14101. doi:10.1073/pnas.0403520101 15375211 PMC521125

[197] Hale C, Kleppe K, Terns RM, Terns MP. 2008. Prokaryotic silencing (psi)RNAs in Pyrococcus furiosus. RNA 14:2572–2579. doi:10.1261/rna.1246808 18971321 PMC2590957

[198] Jäger D, Förstner KU, Sharma CM, Santangelo TJ, Reeve JN. 2014. Primary transcriptome map of the hyperthermophilic archaeon Thermococcus kodakarensis. BMC Genomics 15:684. doi:10.1186/1471-2164-15-684 25127548 PMC4247193

[199] Klein RJ, Misulovin Z, Eddy SR. 2002. Noncoding RNA genes identified in AT-rich hyperthermophiles. Proc Natl Acad Sci USA 99:7542–7547. doi:10.1073/pnas.112063799 12032319 PMC124278

[200] Phok K, Moisan A, Rinaldi D, Brucato N, Carpousis AJ, Gaspin C, Clouet-d’Orval B. 2011. Identification of CRISPR and riboswitch related RNAs among novel noncoding RNAs of the euryarchaeon Pyrococcus abyssi. BMC Genomics 12:312. doi:10.1186/1471-2164-12-312 21668986 PMC3124441

[201] Toffano-Nioche C, Ott A, Crozat E, Nguyen AN, Zytnicki M, Leclerc F, Forterre P, Bouloc P, Gautheret D. 2013. RNA at 92°C: the non-coding transcriptome of the hyperthermophilic archaeon Pyrococcus abyssi. RNA Biol 10:1211–1220. doi:10.4161/rna.25567 23884177 PMC3849170

[202] Bräsen C, Esser D, Rauch B, Siebers B. 2014. Carbohydrate metabolism in Archaea: current insights into unusual enzymes and pathways and their regulation. Microbiol Mol Biol Rev 78:89–175. doi:10.1128/MMBR.00041-13 24600042 PMC3957730

[203] Wenck BR, Santangelo TJ. 2020. Archaeal transcription. Transcription 11:199–210. doi:10.1080/21541264.2020.1838865 33112729 PMC7714419

[204] Koga Y. 2012. Thermal adaptation of the archaeal and bacterial lipid membranes. Archaea 2012:789652. doi:10.1155/2012/789652 22927779 PMC3426160

[205] Moody ERR, Mahendrarajah TA, Dombrowski N, Clark JW, Petitjean C, Offre P, Szöllősi GJ, Spang A, Williams TA. 2022. An estimate of the deepest branches of the tree of life from ancient vertically evolving genes. eLife 11:e66695. doi:10.7554/eLife.66695 35190025 PMC8890751

[206] Hug LA, Baker BJ, Anantharaman K, Brown CT, Probst AJ, Castelle CJ, Butterfield CN, Hernsdorf AW, Amano Y, Ise K, Suzuki Y, Dudek N, Relman DA, Finstad KM, Amundson R, Thomas BC, Banfield JF. 2016. A new view of the tree of life. Nat Microbiol 1:16048. doi:10.1038/nmicrobiol.2016.48 27572647

[207] Catchpole RJ, Forterre P. 2019. The evolution of reverse gyrase suggests a nonhyperthermophilic last universal common ancestor. Mol Biol Evol 36:2737–2747. doi:10.1093/molbev/msz180 31504731 PMC6878951

[208] Adam PS, Borrel G, Brochier-Armanet C, Gribaldo S. 2017. The growing tree of Archaea: new perspectives on their diversity, evolution and ecology. ISME J 11:2407–2425. doi:10.1038/ismej.2017.122 28777382 PMC5649171

